# Pharmacological read-through of nonsense ARSB mutations as a potential therapeutic approach for mucopolysaccharidosis VI

**DOI:** 10.1007/s10545-012-9521-y

**Published:** 2012-09-13

**Authors:** Rosa Bartolomeo, Elena V. Polishchuk, Nicola Volpi, Roman S. Polishchuk, Alberto Auricchio

**Affiliations:** 1 Telethon Institute of Genetics and Medicine (TIGEM), Via P. Castellino 111, 80131 Naples, Italy; 2 Medical Genetics, Department of Pediatrics, “Federico II” University, Naples, Italy; 3 Department of Biology, University of Modena & Reggio Emilia, Modena, Italy

## Abstract

Mucopolysaccharidosis type VI (MPS VI) is a severe lysosomal storage disorder without central nervous system involvement caused by arylsulfatase B (ARSB) deficiency. MPS VI is characterized by dysostosis multiplex, corneal clouding, heart valve defects and urinary excretion of glycosaminoglycans (GAGs). The current treatment for MPS VI is enzyme replacement therapy (ERT) which has limited efficacy on bone, joints and heart valve disease, as well as high costs. A potential therapeutic approach for the subgroup of MPS VI patients that carry nonsense mutations is to enhance stop-codon read-through, using small molecules, to restore production of the full-length ARSB protein. In this study we investigated whether two compounds known to induce stop codon read-through, the aminoglycoside gentamicin and PTC124, can promote read-through of four different ARSB nonsense mutations (p.R315X, p.R327X, p.Q456X and p.Q503X) associated with MPS VI and enable the synthesis of full-length functional ARSB protein in patients fibroblast cell lines. Our study demonstrates that PTC124 but not gentamicin, increases the level of ARSB activity in three MPS VI patient fibroblast cell lines. In two of them the levels of ARSB activity obtained were significantly higher than in untreated cells, reaching ≤2.5 % of those detected in wild-type fibroblasts and resulting in significant reduction of lysosomal size. Since even small increases in enzyme activity can dramatically influence the clinical phenotype of MPS VI, our study suggests that pharmacological read-through may be combined with ERT potentially increasing therapeutic efficacy in those patients bearing nonsense ARSB mutations.

**Electronic supplementary material:**

The online version of this article (doi:10.1007/s10545-012-9521-y) contains supplementary material, which is available to authorized users.

## Introduction

Mucopolysaccharidosis VI (MPS VI; Maroteaux-Lamy syndrome; OMIM #253200) is an autosomal recessive lysosomal storage disorder (LSD), that belongs to the group of mucopolysaccharidoses (MPS). The MPS are caused by defects in lysosomal enzymes that results in widespread intra- and extra-cellular accumulation of glycosaminoglycans (GAGs). MPS VI is caused by deficiency of the enzyme arylsulfatase B [ARSB] (N-acetylgalactosamine-4-sulfatase; EC 3.1.6.12) which removes the C4 sulfate ester group from the N-acetylgalactosamine sugar residue at the nonreducing terminus of the glycosaminoglycans dermatan sulfate and chondroitin sulfate (Neufeld and Muenzer 2001). Deficiency of ARSB results in the intralysosomal storage and urinary excretion of these partially degraded GAGs. The ARSB gene encodes for a polypeptide precursor of 533 amino acids, with a molecular weight of 55.8 kDa that is translocated into the lumen of the endoplasmic reticulum. Subsequently the protein is glycosylated (66 kDa) and then processed into a mature form (57 kDa) (Litjens et al 1989).

ARSB is secreted from normal cells, up taken by adjacent cells and directed to the lysosomal compartment through binding to the mannose-6 phosphate receptor (MPR) present on the plasma and endosomal membranes of most cell types (Dahms et al 1989). The enzyme reuptake by MPR that results in cross-correction of ARSB-deficient cells represents the basis for the current treatment strategies: the bone marrow/hematopoietic stem cell transplantation and the enzyme replacement therapy (ERT) (Valayannopoulos et al 2010). However, each of these treatments presents limitations and therapies that efficiently correct the bone, heart and central nervous system (CNS) anomalies of MPS are currently unavailable.

Therefore, there is need for more effective therapeutic strategies for MPS VI. Gene therapy, for instance, is aiming at converting liver in a “factory” for sustained systemic release of ARSB following a single intravascular administration of therapeutic viral vectors (Cotugno et al 2011). However, the safety and efficacy of this strategy has yet to be demonstrated in humans. Another therapeutic option can be used in MPS VI patients that bear premature stop-codon (PTC) mutations. The approach aims at suppressing the effect of the PTC by inducing its translational read-through (Manuvakhova et al 2000). Indeed, it has recently been demonstrated that certain low-molecular-weight drugs can influence the fidelity of stop codon recognition and restore production of full-length proteins (Linde and Kerem 2008). The two read-through inducer drugs that are most widely used are the aminoglycoside gentamicin and PTC124. Gentamicin antimicrobial effect is exerted by binding to polysomes and interfering with protein synthesis causing misreading and premature termination of mRNA translation (Brunton et al 2006). Gentamicin-enhanced stop codon read-through has been used as a therapeutic strategy for genetic diseases, including cystic fibrosis, Duchenne muscular dystrophy and ataxia-teleangectasia (Howard et al 2000; Du et al 2002; Lai et al 2004). Nevertheless, limitations on the use of gentamicin include its relatively low cell permeability and its toxic side effects (e.g., kidney damage and hearing loss). PTC therapeutics has recently developed a new, more-efficient read-through inducing drug, known as PTC124. PTC124 is an orally-bioavailable small molecule compound that promotes dose-dependent suppression of premature translation termination without concomitant effects on normal termination or mRNA decay. Various Phase II and Phase II-III clinical trials to prove the safety of this compound have been completed (http://clinicaltrials.gov/ct2/results?term=ataluren). PTC-124 has high permeability and unlike aminoglycosides, it has not been associated with serious toxic side effects (Hirawat et al 2007). PTC124 probably acts at a ribosomal location different from the one used by aminoglycosides since it is part of a structurally distinct class of drugs (Hirawat et al 2007).

Approximately 33 % of inherited or acquired diseases are due to PTCs (Mendell and Dietz 2001). PTCs have been identified in a large cohort of patients with MPS (Brooks et al 2006); in particular, ARSB mutational analysis in MPS VI patients has allowed to identify 16 different ARSB nonsense mutations (Human Gene Mutation Database; http://www.hgmd.cf.ac.uk/ac/index.php, last access on January 2012) which can be potential targets of stop codon read-through. However, so far, the only evidence that supports the efficacy of stop codon read-through for MPS has been in cells from MPS I patients with PTC mutations. Gentamicin treatment of these cells resulted in increase iduronidase activity, up to 3 % of wild-type enzyme levels, which reduced substrate storage (Keeling et al 2001).

In the current study we investigated whether gentamicin and PTC124 have the potential to read-through ARSB PTCs and increase enzyme activity in MPS VI patients cell lines. Our results indicate that PTC124-mediated stop codon read-through significantly increases ARSB activity which results in reduction of GAG storage as demonstrated by the significant decrease in lysosomal size observed in drug-treated MPS VI cells.

## Results

### Reduction of baseline ARSB activity levels in MPS VI cell lines for more sensitive measurement of PTC read-through

To test the feasibility of stop codon read-through for MPS VI, we used four MPS VI primary fibroblast cell lines (Table 1), cells from a normal individual and cells from an MPS VI patient (ML5), without PTCs and thus not susceptible to read-through, as control.

**Table 1:** MPS VI patient fibroblasts cell lines

Patient ID	Nonsense mutation					
	Amino acid substitution	Nucleotide substitution	Stop codon context	Exon	Second mutation	Phenotype
ML1	R315X	c.943 C > T	cuu**UGA**gga	V	Del exon V	Severe
ML2	R327X	c.979 C > T	guc**UGA**ggg	V	R327X	Severe
ML3	Q456X	c.1366 C > T	ucu**UAA**uac	VIII	C447S	Severe
ML4	Q503X	c.1507 C > T	cua**UAG**uuc	VIII	Q503X	Intermediate
	Mutation	Second Mutation		Comments		
ML5	c.1213 + 6 T > C	c.1213 + 6 T > C		Alternative splicing		Intermediate

Stop codon read-through can result in subtle increments in protein levels therefore it is essential to have a cellular system in which pre-treatment gene product levels are constant and predictable. During the initial stage of this study we observed that this is not the case for ARSB enzymatic activity in MPS VI primary fibroblast cell lines; we hypothesised that this may be due to ARSB uptake from culture medium, thus resulting in variable baseline ARSB activity levels. For example, MPS VI cells that bear mutations causing a severe phenotype and for which the expected ARSB levels should be below the detection limit of the enzymatic assay, routinely exhibited ARSB activity levels ranging between 1.5 % and 6 % of wild-type ARSB activity.

To test if there is uptake from the medium and to reduce the baseline ARSB activities, MPS VI primary fibroblasts were maintained in culture medium with either 2.5 mM or 5 mM mannose-6-phosphate (M6P), in order to saturate MPRs and to inhibit exogenous ARSB uptake from the medium (Supplementary Fig. 1a). The levels of endogenous ARSB activity from MPS VI cells treated with M6P were lower than those from untreated cells and were almost undetectable in the presence of 5 mM M6P. Thus, all experiments have been performed in the presence of 5 mM M6P. MPRs are required for the normal production of lysosomal enzymes, as well as their proper maturation and intracellular delivery to lysosomes (Dahms et al 1989), thus we assessed if culturing primary fibroblasts in the presence of M6P interferes with lysosomal enzymes processing. We tested the activity of two additional lysosomal enzymes in MPS VI cells cultured or not in the presence of M6P. We did not observe any significant differences in the activity of β-galactosidase and α-1-4-glucosidase between cells treated or not with M6P (Supplementary Fig. 1b), suggesting that M6P addition does not interfere with normal intracellular lysosomal enzymes processing.

### Assessment of ARSB mRNA levels in MPS VI cell lines before addition of read-through inducing compounds

Because transcripts carrying PTCs represent the template for read-through treatment, it was hypothesized that nonsense-mediated mRNA decay (NMD) might impair the read-through response (Kuzmiak and Maquat 2006). Consequently, analyzing the levels of mRNA before setting up a read-through treatment may be instrumental to identify patients with highest potential to respond to the treatment. The NMD pathway is known to cause the degradation of mRNAs that carry a PTC in a position 50 nucleotides upstream of the last exon–exon junction (Nagy and Maquat 1998). However, because there are exceptions to this rule (Linde and Kerem 2008) and exceptions are not usually predictable, it is advisable to confirm experimentally if a PTC-bearing mRNA is an NMD target.

**Fig. 1:**
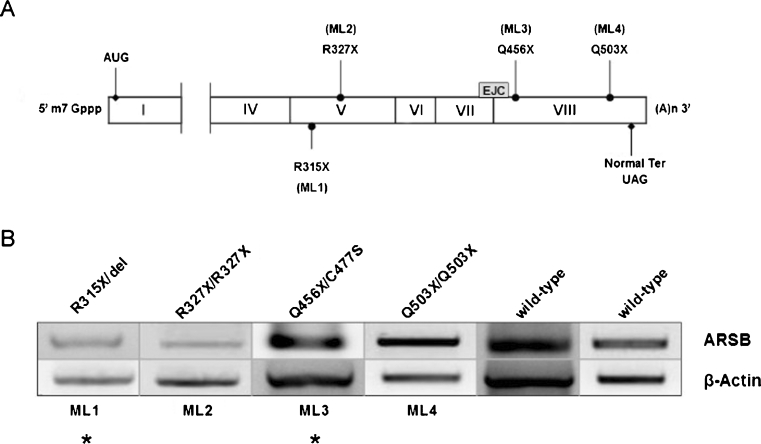
**a** Schematic representation of the ARSB PTC mutations present in the MPS VI cell lines. EJC: exon-junction complex. (ML1), (ML2), (ML3) and (ML4): MPS VI cell lines with corresponding PTCs. **b** ARSB mRNA levels in MPS VI cell lines. RT-PCR reactions were performed on total RNA to amplify: i) a 632 bp ARSB cDNA fragment (from exon 7 to the 3′-UTR) from ML1, ML2, ML4 and wild-type (non-MPS VI individuals); ii) a 377 bp cDNA fragment (from exon 8 to the 3′-UTR) from ML3; iii) a 839 bp β-Actin fragment, as control. The resulting PCR products were separated on a 1 % agarose gel. Lanes marked with (*asterisk*) correspond to two consecutive PCR rounds of amplifications performed on the same template

Our analysis included mutant transcripts predicted to escape NMD (ML3 and ML4) as well as two ARSB transcripts (ML1 and ML2) expected to be subject to NMD based on previous reports (Fig. 1a) (Garrido et al 2008).

Our RT-PCR results shown in Fig. 1b confirm that NMD of the mutant ARSB transcripts can be both PTC-position dependent and independent. Indeed, we found high (ML4 cell line) and low (ML1 and ML2 cell lines) ARSB mRNA levels, as one would predict from the position of the corresponding PTC (Fig. 1a). In the ML3 cell line, where the PTC position is not predicted to trigger NMD, we observed transcript levels lower than expected (please note that the band in the ML3 lane in Fig. 1b results from two consecutive cycles of PCR amplification) suggesting that susceptibility of the transcripts to NMD predicted on the basis of the PTC position should be regarded only as an indication and should be verified in experimental conditions. In conclusion, we found that before addition of read-through inducing compounds all MPS VI cell lines are potentially responsive to treatment, as we found in all of them detectable levels of ARSB transcript spared by the NMD process. However, the presence of ARSB transcript is necessary but not sufficient for drug-induced read-through to occur since factors like: i) the stop codon context sequence; ii) the sensitivity of the detection system and iii) the cell culture conditions can dramatically influence the read-though efficiency.

As treatment with neither gentamicin nor PTC124 has been reported to affect mRNA levels (Welch et al 2007; Du et al 2008), we did not perform ARSB transcript level analysis following incubation with the drugs.

### Treatment with PTC124 but not with gentamicin significantly increases ARSB enzymatic activity and results in GAG clearance in MPS VI cell lines

To test the efficacy of read-through induction, we selected two known read-through inducing compounds, gentamicin (1 mg/ml) and PTC124 (3.3 μM and 10 μM), and one incubation time point (48 h). The drug doses were selected based on previously published data (Keeling et al 2001; Welch et al 2007). As time points we tested 24, 48 and 72 h after drug administration and found that the highest increase in enzymatic activity is observed at 48 h (data not shown).

**Fig. 2:**
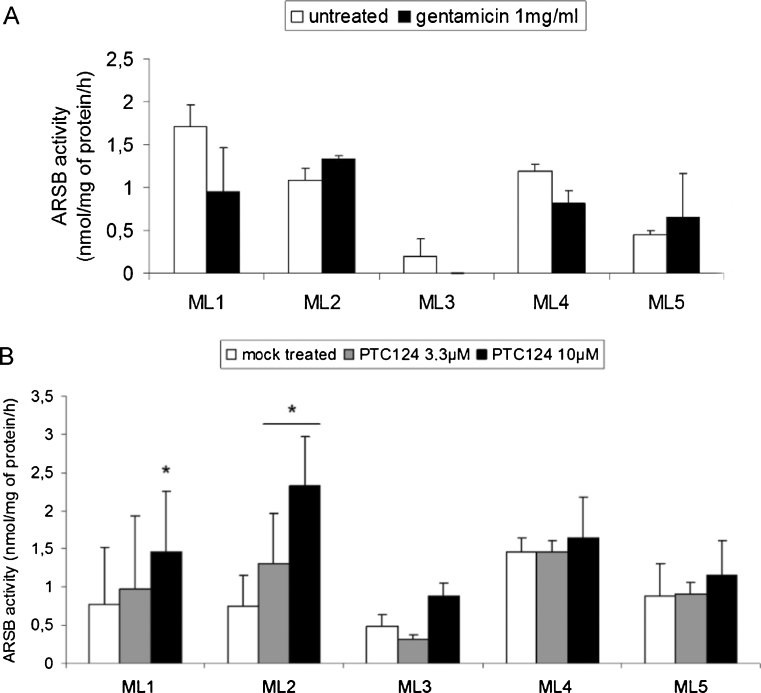
**a** Gentamicin does not induce significant PTCs read-through in MPS VI fibroblasts. ML1 (passage 6), ML2 (passage 10), ML3 (passage 9), ML4 (passage 14) and ML5 (passage 8) fibroblasts were cultured in the presence of 5 mM M6P for 7–13 days and then incubated or not for 48 h with 1 mg/ml gentamicin. Data are shown as average + standard error (SE) (two independent experiments). **b** PTC124-mediated stop codon read-through in MPS VI fibroblasts. ML1 (passage 6), ML2 (passage 10), ML3 (passage 9), ML4 (passage 14) and ML5 (passage 8) fibroblasts were cultured in the presence of 5 mM M6P for 7–13 days and then incubated or not for 48 h with 3.3 μM PTC124, 10 μM PTC124 or 1.6 % DMSO (mock treated). Data are shown as average + SE of two independent experiments for ML3, ML4 and ML5 cells; data from ML1 and ML2 cells are from three independent experiments. * *P* ≤ 0.05 indicates the statistical difference between PTC124- and DMSO-treated cells. Additional *t*-test values information are found in Supplementary Table 1

As shown in Fig. 2a, although the ARSB background activity has not been completely abolished by the incubation with M6P, and some variability is still observed among the different cell lines, no recovery of ARSB activity was observed following incubation with gentamicin in the four MPS VI cell lines used. Notably, we were not able to perform experiments on early culture passages for any of the MPS VI cell lines and this may have been critical when using gentamicin. Indeed, recent studies have reported that membrane permeability to aminoglycosides varies significantly between different cell types and culture passages; particularly, the permeability or efflux of gentamicin in cultured primary fibroblasts is reduced with increasing the cell passage number (Schroeder et al 1984; Keeling et al 2001), suggesting that the primary fibroblast cell line passage is crucial for gentamicin activity. Thus it is possible that the culture passage at which our MPS VI cell lines were tested for gentamicin-induced suppression of PTCs was not optimal to detect its effect. Besides, to overcome the reduced membrane permeability we used the highest gentamicin concentration which has been reported in the literature (Manuvakhova et al 2000). However, gentamicin treatment at this concentration appears to be toxic since all cell lines, except those derived from patient ML2, present lower ARSB activity in the presence than in the absence of gentamicin (Fig. 2a).

After PTC124 treatment, increased ARSB activity was detected in all but one MPS VI patient fibroblast cell lines (Fig. 2b). In particular, we found that PTC124 treatment significantly increased ARSB activity of ML2 fibroblasts: to 2.32 nmol/mg of protein/h (PTC124 [10 μM]) and 1.31 nmol/mg of protein/h (PTC124 [3.3 μM]), thrice and twice the levels of untreated cells (0.74 nmol/mg of protein/h), respectively. In ML1 and ML3 fibroblasts the increase in ARSB activity was detected only with the highest PTC124 concentration (10 μM) and, even if in both cell lines the ARSB activity almost doubled in treated compared to untreated control cells, the increase was significant only in ML1 cells (Supplementary Table 1). Finally, treatment with PTC124 was ineffective in both the ML4 line and, as expected, in the control ML5 cell line. Notably, ML1 and ML2 cell lines carry PTCs with the highest read-through potential (Manuvakhova et al 2000), which inversely correlates with the translation-termination efficiency, i.e., UGA has the highest read-through efficiency, followed by UAG and, to a lesser extent, UAA (Manuvakhova et al 2000; Howard et al 2000).

Since PTC-read-through induces the insertion of random aminoacidic residues at the stop codon position, some of the resulting full-length proteins will be wild-type and retain catalytic activity while some will contain aminoacidic substitutions which might impair catalytic activity. Since the latter will not be detected by the ARSB enzymatic assay, we analyzed the same cellular extracts used for the ARSB enzymatic assay by Western blot analysis with anti-ARSB antibodies (data not shown). Western blot analysis should allow semi-quantitative measurements of ARSB protein independently of its enzymatic activity. We were not able to detect any increase in the levels of ARSB protein in cells treated with PTC124 compared to untreated cells (data not shown). However, given the low-resolution of the Western blot, we cannot exclude that this might have hampered the detection of low levels of ARSB protein produced in PTC124-treated MPS VI cells.

**Fig. 3:**
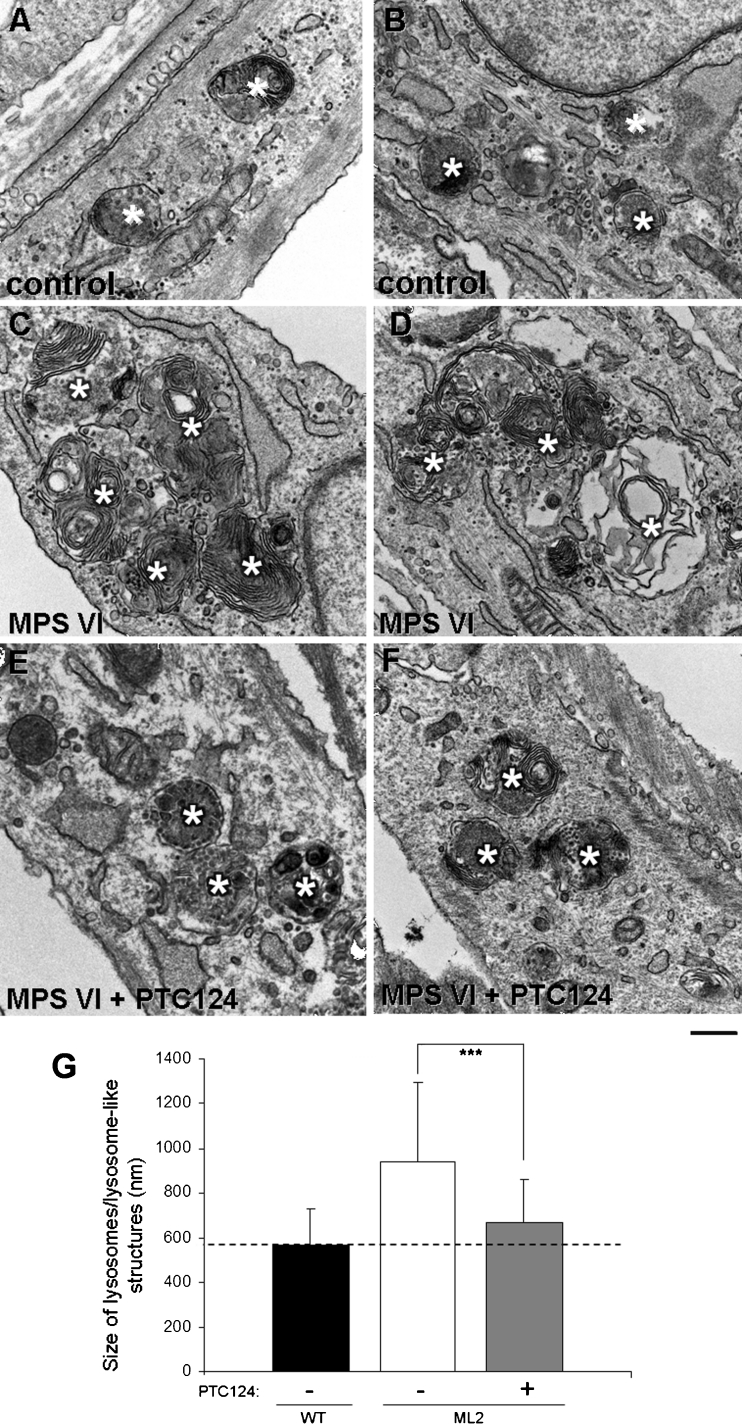
Treatment with PTC124 reduces the size of lysosome-like organelles in ML2 fibroblasts. Wild-type (control, WT) and ML2 fibroblasts were cultured in the presence of 5 mM M6P for 10 days and then ML2 cells were incubated with 10 μM PTC124 or 1.6 % DMSO (mock treated) for 48 h, then fixed and prepared for EM as described in Materials and Methods. *Asterisks* in each image indicate the center of lysosome-like organelles. Panels **a** and **b** show representative lysosomes in wild-type fibroblasts. ML2 fibroblasts exhibit numerous large lysosome-like structures (**c, d**). The same cells treated with PTC124 show lysosome-like structures of reduced size (**e, f**). Graph in **g** represents quantification of lysosome size (100 structures of lysosomal origin were analyzed for each condition). The data is expressed as the mean ± SD, while *** *P* < 0.001 indicates the statistical difference between PTC124- and DMSO-treated ML2 cells. *Scale bar*, 500 nm (nm: nanometers)

To further explore if the ARSB activity levels achieved after PTC124 treatment can reverse abnormal GAG storage, we performed electron microscopy (EM) analysis of both the MPS VI cell line 2 (ML2), the best responder to PTC124, and wild-type fibroblasts as control (Fig. 3a, b). ML2 cells were incubated at the highest PTC124 concentration (10 μM) for 2 and 10 days. As shown in Fig. 3 (panels c, d), fibroblasts from ML2 patient exhibit numerous large lysosome-like structures whose size is significantly reduced after PTC124 treatment (panels e, f, g); this trend to normalization observed after 2 days of incubation with PTC124 is retained after 10 days of treatment (data not shown). These data support the efficacy of PTC124 at reducing lysosomal storage and engulfment, one of the main features of MPS VI.

## Discussion

A nonsense mutation prematurely halts the synthesis of a protein: stop codon read-through is being considered as a potential therapeutic strategy (either alone or in combination with other therapies) for recessive genetic diseases due to nonsense mutations. Indeed several studies, most of which performed in vitro, have shown its efficacy (Linde and Kerem 2008).

For this purpose, the two drugs that are most widely used and most promising are the aminoglycoside gentamicin and the investigational new drug PTC124 (Welch et al 2007). Indeed, both are able to induce a beneficial stop codon read-through based on their ability to alter the ribosomal proofreading activity on premature termination codons, while remaining ineffective on bona fide termination codons (Rospert et al 2005). There are ongoing clinical studies testing the safety and efficacy of both, however, PTC124 may be preferable as its use is not associated with the toxicity observed with prolonged administrations of aminoglycosides. Notably, our study is one of the first which tests the effect of PTC124 in LSD (Sarkar et al 2011), as only in one additional report it has been shown that aminoglycosides can suppress nonsense mutations within the iduronidase gene mutated in MPS I (Keeling et al 2001; Wang et al 2012). A critical issue when considering this therapeutic strategy is to establish whether the efficacy of the stop codon read-through may be clinically relevant. LSDs, including MPS VI, offer a unique opportunity to address this as there is a well-defined correlation between residual enzymatic activity and disease severity: 5–10 % of enzymatic activity is thus considered therapeutic in several LSDs (Neufeld and Muenzer 2001). In addition, since the chemical drugs that are used to influence stop codon read-through tend to be lower in molecular weight and more diffusible than the proteins used for ERT, it is possible that read-through drugs will be able to reach sites of disease like bone, cartilage or brain that are resistant to the conventional ERT. Since PTCs are generally associated with low residual enzymatic activity and severe clinical manifestations, a therapy that combines both ERT and stop codon read-through may result in maximum substrate clearance and a more effective therapy for MPS VI patients with severe symptoms (Brooks et al 2006).

Importantly, we set out to determine the potential of pharmacological read-through on intact cells by analyzing the effects of gentamicin and PTC124 on endogenous nonsense-bearing transcripts directly, assuming that the read-through effect could be quantified as an increase in ARSB enzymatic activity compared to that detected in control untreated cells. Although we were able to detect significant increases in ARSB activity in MPS VI cells treated with PTC124, the levels reached did not exceed 3 % of wild-type. One possibility to explain these low levels, which however are similar to those achieved by others in MPS I cell lines, is that measuring ARSB activity may not be sensitive enough to detect low increases, since cultured MPS VI fibroblasts can uptake exogenous ARSB from the culture medium and this might result in variable degrees of baseline ARSB activity measured in control untreated fibroblasts. This might partially mask read-through-induced increases in ARSB activity after treatment with read-through agents, especially if this induction occurs at low levels. Also, we cannot exclude that culturing cells with M6P to block the uptake of exogenous ARSB, despite not impacting the activity levels of two lysosomal enzymes different from ARSB, might alter intracellular pathways involved in lysosomal enzyme processing, possibly having an effect on read-through efficacy.

Importantly, we show that PTC124-induced increase of ARSB activity levels to 2.5 % of normal significantly reduces lysosomal size suggesting that PTC124 induces clearance of lysosomal storage. This further supports the concept that slight increases in enzymatic activity can have a profound impact on LSDs.

Altogether, our results suggest that PTC124-mediated stop codon read-through has the potential to attenuate both the biochemical and the morphological abnormalities of MPS VI. Thus, PTC124 use could be considered in combination with ERT to enhance therapeutic efficacy in those districts like bone or cartilage which could be reached more effectively by a small molecule drug than by a larger protein.

## Material and methods

### Cell culture

Fibroblasts from MPS VI patients ML2, ML4 and ML5 were provided by the Telethon Cell line and DNA Bank from Patients with Genetic Diseases (Dr Mirella Filocamo, Gaslini Hospital Genoa, Italy). Fibroblasts from MPS VI patient ML3 were provided by the Children Hospital, University of Mainz, Germany. ML1 MPS VI and normal control fibroblasts were available at the Department of Pediatrics, Federico II University, Naples, Italy.

All cell lines were grown at 37 °C with 5%CO2, in Dulbecco’s modified Eagle’s medium (DMEM, Celbio, Milan, Italy) supplemented with 20 % fetal bovine serum (FBS, Gibco, Invitrogen corporation, Carlsbad, CA, USA), 100U/ml penicillin and 100 μg/ml streptomycin (Sigma-Aldrich, Milan, Italy). All experiments were conducted on fibroblasts at 50–70 % confluence and at various passages (specified in the Results section). We performed three independent replicates of those experiments in which drug treatment resulted in increased enzymatic activity (PTC124 treatment of ML1 and ML2 cell lines), and duplicates of those without significant enzymatic increase following addition of read-through inducing compounds.

### Reverse transcriptase-PCR

Fibroblasts were grown until confluence. Cells were washed twice with Phosphate Buffer Saline (PBS, Gibco, Invitrogen Corporation, Carlsbad, CA, USA) and harvested using trypsin/EDTA (Gibco, Invitrogen Corporation, Carlsbad, CA, USA) for 3–5 min; then the cell suspension was centrifuged at 1200 g for 5 min. The cell pellet was then washed with PBS and separated by centrifugation. The cell pellet was used to isolate total RNA using the QIAshredder and the RNeasy Minikit (Qiagen, Hilden, Germany). RNA was quantified with a Nanodrop apparatus and analyzed on an agarose gel.

A reverse transcription reaction was carried out from 1 μg of total RNA using the SuperScript® III Reverse Transcriptase (RT) (Invitrogen corporation, Carlsbad, CA, USA), following the manufacturer’s recommendations. To amplify an ARSB cDNA fragment, RT-PCR was carried out using the following primers: forward 5′- CAT GGC TCC AGC AAA GGA T – 3′ and reverse 5′ – GTC CCA AGG CTT AGG AAA G – 3′ (product length of 632 bp). For ML3 fibroblasts, forward 5′-GGT TCC CTC CAC CGT CTT – 3′ and reverse 5′-GCCTGAGGTCCAACTTCC-3′ primers were used to amplify an ARSB cDNA fragment (375 bp) specific for the PTC-bearing allele.

β-actin was also amplified using the following primers: forward 5′ – GTC CCA AGG CTT AGG AAA G – 3′ and reverse 5′ – GTC CCA AGG CTT AGG AAA G – 3′ (product length of 839 bp).

PCRs were performed using TaQ Gold DNA polymerase (Roche, Indianapolis, IN, USA) and consisted of 1 μl of template cDNA, 1.25 U TaQ Gold DNA polymerase, 2.5 mM MgCl2 in the recommended buffer, 200 μM of dNTPs, and 0.5 μM of each primer in a final volume of 25 μl. The reactions cycles used are the following: 5 min at 95 °C; 40 cycles of denaturation at 94 °C for 45 s, annealing at 56 °C for 45 s and elongation at 72 °C for 85 s; final extension step at 72 °C for 7 min. The amplification reactions were performed in a T3000 Thermocycler (Biometra, Goettingen, Germany). The resulting PCR products were separated on a 1 % agarose gel. Samples from patients ML1 and ML3 required two consecutive rounds of PCR amplifications performed on the same template.

### Gentamicin and PTC124 treatment

All the MPS VI cell lines were maintained in culture medium containing 5 mM M6P, for a period of 7–13 days before and during the incubation with the drugs.

Read-through induction cells were incubated in the presence of 1 mg/ml gentamicin (Sigma-Aldrich, Milan, Italy) for 12, 24, 48 or 72 h. Stock solutions (6 mM) of PTC124 (Selleck Chemical LLC, Houston, USA) were prepared in 100 % DMSO (Sigma-Aldrich, Milan, Italy). Fibroblasts were incubated in the presence of 3.3 μM, 10 μM PTC-124 or 1.6 % DMSO (mock treated) for 48 h. During the incubation time, cells were cultivated in the absence of antibiotic drugs.

### Protein extraction and ARSB activity assay

After the selected time point, cells were washed twice with PBS and harvested using trypsin/EDTA for 3–5 min; then the cell suspension was centrifuged at 1200 g for 5 min. The cell pellet was then washed twice with PBS and then re-centrifuged. The resulting supernatant was removed and cells were lysed in water in the presence of a protease inhibitor cocktail (Roche, Indianapolis, USA) with three repeated cycles of freezing and thawing; cell debris were eliminated by centrifugation at 13,000 rpm for 20 min; the supernatant was collected and subjected to further analysis. Total protein concentration in cellular extracts was measured using the colorimetric BCA protein assay kit (Pierce Chemical Co, Boston, MA, USA) and a standard curve with seven different concentrations of bovine serum albumine (Pierce Chemical Co, Boston, MA, USA): 0.05, 0.1, 0.2, 0.4, 0.6, 0.8 and 1 μg/μl. Colorimetric reaction was quantified with an absorbance reader (Infinite F200, Tecan, Mannedorf, Switzerland) using a 560 nm filter.

The ARSB activity assay was performed as described previously (Tessitore et al 2008). Briefly, 15 ug of proteins were incubated with 40 μl of the fluorogenic substrate, 4-methylumbelliferyl-sulfate potassium salt (12.5 mM Sigma-Aldrich, Milan, Italy), for 3 h at 37 °C in the presence of 40 μl of silver nitrate (0.75 mM, Carlo Erba, Milan, Italy), which is known to inhibit the activity deriving from other sulfatases. The reaction was stopped by adding 200 μl of the carbonate stop buffer (0.5 M NaHCO3/0.5 M Na2CO3, pH 10.7), and the fluorescence of the 4-methylumbelliferone liberated was measured in a fluorimeter (Infinite F200, Tecan, Mannedorf, Switzerland) using 365 nm excitation and 460 nm emission. The enzyme activities were calculated using a standard curve of the fluorogenic product, 4-methylumbelliferone (Sigma-Aldrich, Milan, Italy). β-galactosidase and α-glucosidase activities were assayed as previously described, using the appropriate substrates. β-galactosidase (4-methylumbelliferyl-b-D-galactoside, Sigma-Aldrich, Milan, Italy) and α-glucosidase (4-methylumbelliferyl-b-D-glucoside, Sigma-Aldrich, Milan, Italy) (Kresse et al 1982). For cellular extracts the activity is expressed as nmol/mg protein/h.

### Electron microscopy (EM) analysis of MPS VI fibroblasts

Fibroblasts from the ML2 MPS VI patient were treated for 2 days with either PTC124 10 μM or 1.6 % DMSO (mock treated). Wild-type fibroblasts were used as controls. Cells were fixed in 1 % glutaraldehyde in 0.2 M HEPES buffer, post-fixed in uranyl acetate and in OsO_4_. After dehydration through a graded series of ethanol, the cells were embedded in Epoxy resin (Epon 812, Sigma-Aldrich, St. Louis, MO, USA) and polymerized at 60 °C for 72 h. From each sample, thin sections were cut with a Leica EM UC6 ultramicrotome (Leica Mycosystems, Vienna, Austria). EM images were acquired from thin sections using an FEI Tecnai-12 electron microscope (FEI, Eindhoven, Netherlands) equipped with an ULTRA VIEW CCD digital camera (Soft Imaging Systems GmbH, Munster, Germany). Quantification of lysosome-like organelles size was performed using the iTEM software (Soft Imaging Systems GmbH, Munster, Germany).

### Statistical analysis

The statistical analysis of data obtained from each experimental group was performed by using the unpaired two-tail Student’s *t*-test. A *P* value ≤0.05 was considered significant.

## Electronic supplementary material

Below is the link to the electronic supplementary material.

Supplementary Fig. 1 **a** M6P treatment inhibits ARSB uptake from culture medium. ARSB activity measured in ML1 (R315X/del) cells cultured for 10 days in presence of different concentrations of mannose-6-phosphate (M6P). Data are shown as average + SE of three independent experiments. **b**. M6P treatment does not impair lysosomal enzymes activity. β-galactosidase activity and α-1-4-glucosidase activity measured on the same cellular extracts as in 1a (JPEG 8 kb)

High resolution image (TIFF 1.40 MB)

Supplementary Table 1 ARSB activity with and without PTC124 treatment (XLS 24 kb)

## References

[CR1] Brooks DA, Muller VJ, Hopwood JJ (2006). Stop-codon read-through for patients affected by a lysosomal storage disorder. Trends Mol Med.

[CR2] Brunton LL, Gilman A, Goodman LS (2006). Goodman & Gilman’s The pharmacological basis of therapeutics.

[CR3] Cotugno G, Annunziata P, Tessitore A (2011). Long-term amelioration of feline Mucopolysaccharidosis VI after AAV-mediated liver gene transfer. Mol Ther.

[CR4] Dahms NM, Lobel P, Kornfeld S (1989). Mannose 6-phosphate receptors and lysosomal enzyme targeting. J Biol Chem.

[CR5] Du M, Jones JR, Lanier J (2002). Aminoglycoside suppression of a premature stop mutation in a Cftr-/- mouse carrying a human CFTR-G542X transgene. J Mol Med (Berl).

[CR6] Du M, Liu X, Welch EM (2008). PTC124 is an orally bioavailable compound that promotes suppression of the human CFTR-G542X nonsense allele in a CF mouse model. Proc Natl Acad Sci U S A.

[CR7] Garrido E, Cormand B, Hopwood JJ (2008). Maroteaux-Lamy syndrome: functional characterization of pathogenic mutations and polymorphisms in the arylsulfatase B gene. Mol Genet Metab.

[CR8] Hirawat S, Welch EM, Elfring GL (2007). Safety, tolerability, and pharmacokinetics of PTC124, a nonaminoglycoside nonsense mutation suppressor, following single- and multiple-dose administration to healthy male and female adult volunteers. J Clin Pharmacol.

[CR9] Howard MT, Shirts BH, Petros LM (2000). Sequence specificity of aminoglycoside-induced stop condon readthrough: potential implications for treatment of Duchenne muscular dystrophy. Ann Neurol.

[CR10] Keeling KM, Brooks DA, Hopwood JJ (2001). Gentamicin-mediated suppression of Hurler syndrome stop mutations restores a low level of alpha-L-iduronidase activity and reduces lysosomal glycosaminoglycan accumulation. Hum Mol Genet.

[CR11] Kresse H, von Figura K, Klein U (1982). Enzymic diagnosis of the genetic mucopolysaccharide storage disorders. Methods Enzymol.

[CR12] Kuzmiak HA, Maquat LE (2006). Applying nonsense-mediated mRNA decay research to the clinic: progress and challenges. Trends Mol Med.

[CR13] Lai CH, Chun HH, Nahas SA (2004). Correction of ATM gene function by aminoglycoside-induced read-through of premature termination codons. Proc Natl Acad Sci U S A.

[CR14] Linde L, Kerem B (2008). Introducing sense into nonsense in treatments of human genetic diseases. Trends Genet.

[CR15] Litjens T, Baker EG, Beckmann KR (1989). Chromosomal localization of ARSB, the gene for human N-acetylgalactosamine-4-sulphatase. Hum Genet.

[CR16] Manuvakhova M, Keeling K, Bedwell DM (2000). Aminoglycoside antibiotics mediate context-dependent suppression of termination codons in a mammalian translation system. RNA.

[CR17] Mendell JT, Dietz HC (2001). When the message goes awry: disease-producing mutations that influence mRNA content and performance. Cell.

[CR18] Nagy E, Maquat LE (1998). A rule for termination-codon position within intron-containing genes: when nonsense affects RNA abundance. Trends Biochem Sci.

[CR19] Neufeld E, Muenzer J (2001). The mucopolysaccharidoses.

[CR20] Rospert S, Rakwalska M, Dubaquie Y (2005). Polypeptide chain termination and stop codon readthrough on eukaryotic ribosomes. Rev Physiol Biochem Pharmacol.

[CR21] Sarkar C, Zhang Z, Mukherjee AB (2011). Stop codon read-through with PTC124 induces palmitoyl-protein thioesterase-1 activity, reduces thioester load and suppresses apoptosis in cultured cells from INCL patients. Mol Genet Metab.

[CR22] Schroeder F, Goetz I, Roberts E (1984). Age-related alterations in cultured human fibroblast membrane structure and function. Mech Ageing Dev.

[CR23] Tessitore A, Faella A, O’Malley T (2008). Biochemical, pathological, and skeletal improvement of mucopolysaccharidosis VI after gene transfer to liver but not to muscle. Mol Ther.

[CR24] Valayannopoulos V, Nicely H, Harmatz P (2010). Mucopolysaccharidosis VI. Orphanet J Rare Dis.

[CR25] Wang D, Belakhov V, Kandasamy J (2012). The designer aminoglycoside NB84 significantly reduces glycosaminoglycan accumulation associated with MPS I-H in the Idua-W392X mouse. Mol Genet Metab.

[CR26] Welch EM, Barton ER, Zhuo J (2007). PTC124 targets genetic disorders caused by nonsense mutations. Nature.

