# Supplementary figures and images for: Pharmacological read-through of nonsense ARSB mutations as a potential therapeutic approach for mucopolysaccharidosis VI
Source: J Inherit Metab Dis. 2012 Sep 13;36(2):363–71. doi: 10.1007/s10545-012-9521-y (PMC3590409; doi:10.1007/s10545-012-9521-y)

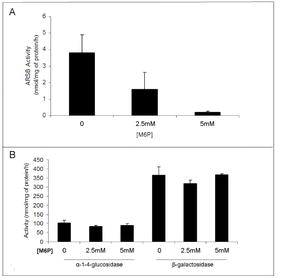

Supplement: Supplementary file 1 — a M6P treatment inhibits ARSB uptake from culture medium. ARSB activity measured in ML1 (R315X/del) cells cultured for 10 days in presence of different concentrations of mannose-6-phosphate (M6P). Data are shown as average + SE of three independent experiments. b. M6P treatment does not impair lysosomal enzymes activity. β-galactosidase activity and α-1-4-glucosidase activity measured on the same cellular extracts as in 1a (JPEG 8 kb) [file 10545_2012_9521_Fig4_ESM.jpg]

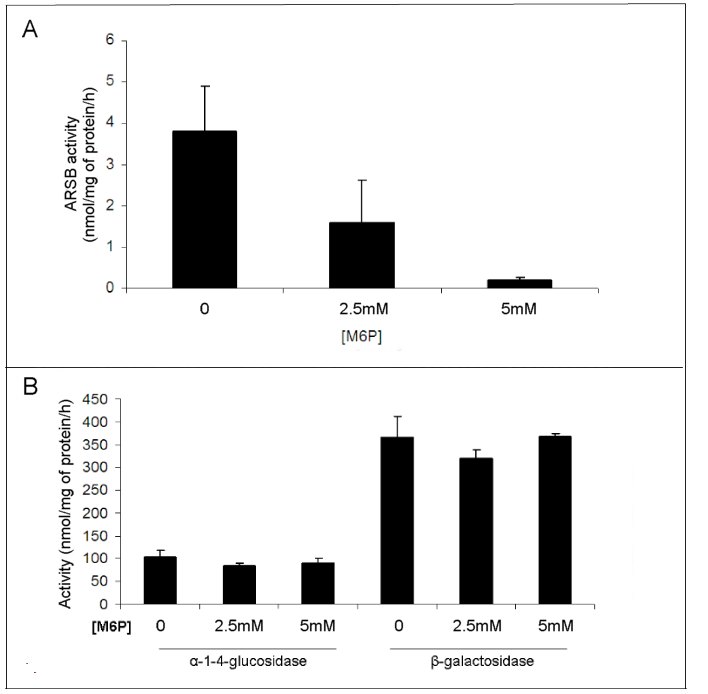

Supplement: Supplementary file 2 — High resolution image (TIFF 1.40 MB) [file 10545_2012_9521_MOESM1_ESM.tif]
